# Micromorphology of the Aristotle’s Lantern in the Sand Dollar, *Scaphechinus mirabilis* (Echinoidea: Echinolampadacea): Insights from Scanning Electron Microscopy and Three-Dimensional X-Ray Microscopy

**DOI:** 10.3390/ani16172733

**Published:** 2026-09-02

**Authors:** Eun Ha Kim, Jung Jun Park, Chang-Moon Lee, Hyeon Jin Kim, Jung Sick Lee

**Affiliations:** 1 Aquaculture Research Division, National Institute of Fisheries Science, Busan 46083, Republic of Korea; 2 School of Biomedical Engineering, Chonnam National University, Yeosu 59626, Republic of Korea; 3 Research Center of Healthcare Biomedical Engineering, Chonnam National University, Yeosu 59626, Republic of Korea; 4 Leading Research Center of Total Solution for Osteoporosis Treatment, Chonnam National University, Yeosu 59626, Republic of Korea; 5 Department of Aqualife Medicine, Chonnam National University, Yeosu 59626, Republic of Korea

**Keywords:** sea urchin, Aristotle’s lantern, structure, SEM, XRM

## Abstract

The morphology and micromorphology of Aristotle’s lantern, the feeding apparatus of *Scaphechinus mirabilis*, were examined using light microscopy, scanning electron microscopy, and X-ray microscopy. *S. mirabilis* had food grooves on the ventral side, which extended to the mouth. Aristotle’s lantern was a star-shaped calcareous skeletal structure attached to the test by the apophysis and consisted of pyramids, joints, and teeth. The pyramid consisted of two demi-pyramids, an epiphysis, and a bump, whereas the joint located between the pyramids consisted of the interpyramidal ligament and rotula. The tooth was an elongated trapezoid with one pointed end (3.98 × 0.97 × 0.38 mm) and was divided into an embedded part and a projection part. The embedded part was connected to the dental ligament and dental promoter muscle, whereas the projection part was divided into a basal projection part covered by the peridental membrane and an apical projection part protruding beyond the tooth slide. The tooth exhibited a calcareous lamellar structure composed of 15–20 layers, and its distal end was worn. These findings indicate that the morphology of the tooth is specialized for internal crushing of sediment and food particles within the Aristotle’s lantern.

## Simple Summary

*Scaphechinus mirabilis* is a sea urchin that inhabits soft substrates and possesses a feeding apparatus called Aristotle’s lantern. In this study, the micromorphology of Aristotle’s lantern in *S. mirabilis* was examined using light microscopy, scanning electron microscopy, and three-dimensional X-ray microscopy. Aristotle’s lantern consisted of skeletal components, including the pyramids, teeth, epiphyses, and rotulae, as well as ligaments and muscles. The teeth were arranged parallel to the body axis along the tooth grooves of the pyramids and were connected to the pyramids by tooth ligaments and muscles. These findings provide morphological information on Aristotle’s lantern in *S. mirabilis* and contribute to a better understanding of the structural diversity of the feeding apparatus in echinoids.

## 1. Introduction

The class Echinoidea comprises 1167 extant species worldwide [1] and is divided into two subclasses, Regularia and Irregularia, based on morphology [2]. Regularia includes sea urchins with a globular test, such as *Anthocidaris crassispina* and *Hemicentrotus pulcherrimus*, and currently comprises 11 orders. Irregularia comprises five orders, including the heart-shaped Spatangoida and the flattened, coin-shaped Echinolampadacea [3].

Echinolampadacea are commonly known as sand dollars because their flattened, coin-like tests become conspicuous following the loss of the surface spines. They are distributed in Australia, Russia, Japan, and Taiwan [4,5,6], as well as along the entire coast of South Korea [7]. *Scaphechinus mirabilis* primarily inhabits sandy or muddy-sand substrates at depths of 3–6 m, occurring either on the sediment surface or burrowed 1–4 cm beneath it [8,9].

Regular echinoids are epifaunal species that primarily inhabit hard substrates in rocky habitats. They protrude Aristotle’s lantern beyond the mouth to graze on macroalgae, sessile or slow-moving benthic invertebrates, and carrion by scraping or fragmenting food items [10]. In contrast, irregular echinoids are infaunal species that inhabit sandy or muddy-sand substrates, where they burrow beneath the sediment surface and feed as deposit feeders on foraminiferans, benthic diatoms, and organic matter [11]. These differences in habitat and feeding strategy are reflected in the morphology and feeding mechanics of Aristotle’s lantern, resulting in distinct morphological and functional adaptations between regular and irregular echinoids [12].

Aristotle’s lantern is a complex musculoskeletal feeding apparatus organized according to the pentameral symmetry of echinoids [13]. In regular echinoids, Aristotle’s lantern typically consists of 40 skeletal ossicles, including five teeth, ten demipyramids, ten epiphyses, five rotulae, and ten compasses. These skeletal elements are interconnected by ligaments and operated by muscles, including the protractor, retractor, and interpyramidal muscles, enabling various movements of the lantern [14]. In contrast, among irregular echinoids, the Aristotle’s lantern of clypeasteroids differs structurally from that of regular echinoids. The clypeasteroid lantern is flattened and non-protrusible, with the teeth oriented horizontally relative to the substrate and functioning in crushing [15,16].

Aristotle’s lantern has been studied from various perspectives. Studies on the Aristotle’s lantern in echinoids have investigated its relative size and functional significance [17], tooth regeneration in *Strongylocentrotus purpuratus* [18], the evolution of the muscular design of the Aristotle’s lantern [14], the presence or absence of the Aristotle’s lantern in Spatangoids [19], and the chemical composition of the test, spines, and Aristotle’s lantern [20]. However, most of these studies have been limited to regular echinoids.

Previous studies on the Aristotle’s lantern in irregular echinoids have investigated its function [21], taxonomic differences in lantern morphology [12], the muscles responsible for tooth movement in *Dendraster excentricus*, *Eucidaris tribuloides*, *S. purpuratus*, and *Echinarachnius parma* [15,22], and the feeding behavior and digestion of *Astriclypeus manni* [11]. However, studies on the morphology and structure of the Aristotle’s lantern in *S. mirabilis* remain limited.

Therefore, this study aimed to characterize the morphology and microstructure of the Aristotle’s lantern in *S. mirabilis* using light microscopy, scanning electron microscopy, and X-ray microscopy. The results provide the first comprehensive anatomical description of the lantern in this species and establish a morphological framework for comparisons with other irregular echinoids.

## 2. Materials and Methods

### 2.1. Specimens

**Figure 1:**
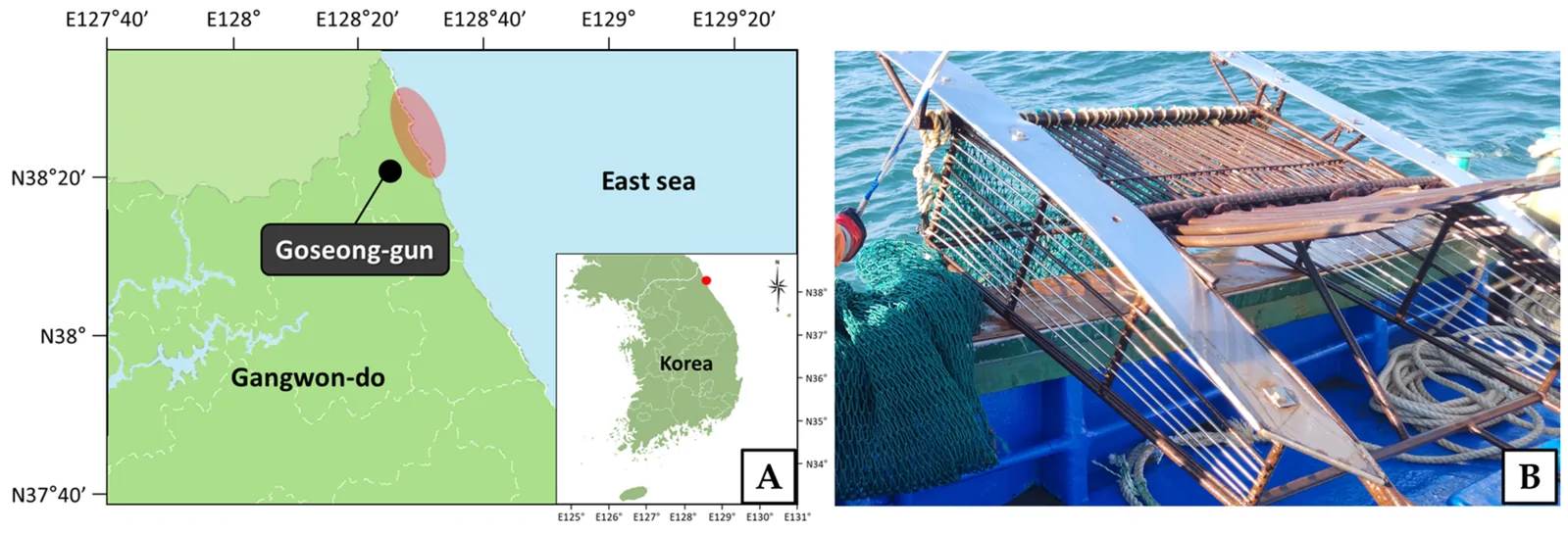
Sampling area (**A**) and dredge net (**B**) used for collecting *Scaphechinus mirabilis*.

A total of 50 individuals of *Scaphechinus mirabilis* were collected using a dredge net at depths of 2–15 m along the northern east coast of the Republic of Korea (38°24′–38°25′ N, 128°28′–128°29′ E) between May and December 2024 (Figure 1). To avoid ontogenetic variation in the morphology of Aristotle’s lantern, only adult individuals (test length ≥ 30 mm) were examined. The minimum adult size was adopted from [23].

### 2.2. Measurements

**Figure 2:**
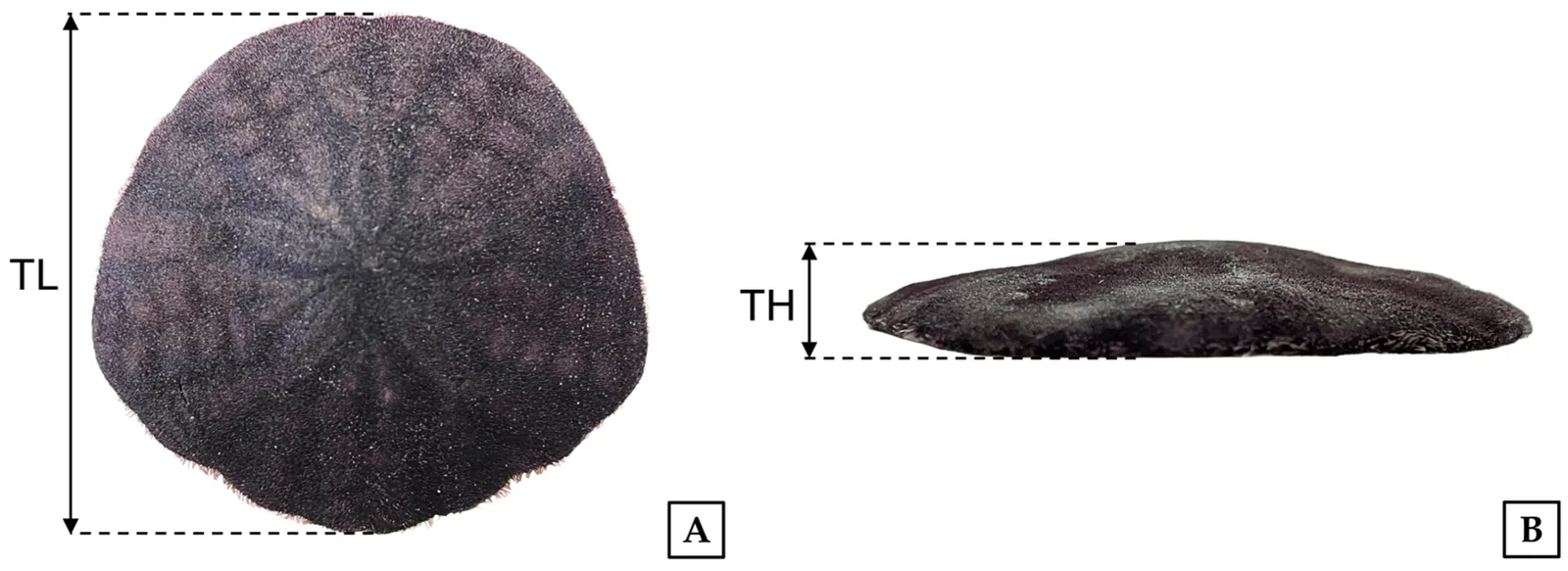
Morphometrics of *Scaphechinus mirabilis*. (**A**): dorsal view. (**B**): lateral view. TH: test height, TL: test length.

Morphometric measurements were performed on 50 individuals (*n* = 50). Test length (TL) and test height (TH) were measured to the nearest 0.01 mm using a digital vernier caliper, and total weight (TW) was measured to the nearest 0.01 g using an electronic balance (Figure 2). The measured values are presented as mean ± standard deviation (SD) and range (minimum–maximum) in Table 1.

### 2.3. Preparation of Ground Specimens

Ground specimens (*n* = 3) were prepared with modifications to the method described in [24]. The Aristotle’s lantern was dissected and immersed in 30% hydrogen peroxide for 24 h to remove the surrounding organic tissues. The cleaned pyramids and teeth were then ground using #320 sandpaper (Noroo Paint & Coatings Co., Anyang, Republic of Korea), rinsed with distilled water, and examined under a stereomicroscope (SZX12, Olympus, Tokyo, Japan).

### 2.4. Light Microscopy

For light microscopy, specimens (*n* = 10) were prepared according to conventional histological procedures, with modifications [25]. A portion of the test was removed, and the specimens were fixed in 10% neutral buffered formalin for 24 h. The fixed specimens were rinsed in running water for 48 h and decalcified in 5% EDTA solution for 48 h. The decalcified specimens were dehydrated through a graded ethanol series, cleared in xylene, and embedded in paraplast (Leica, Wetzlar, Germany). Serial sections approximately 5 μm thick were prepared using a microtome (RM2235, Leica, Wetzlar, Germany). The sections were stained with Mayer’s hematoxylin and 0.5% eosin (H-E) stain, Masson’s trichrome stain, and alcian blue–periodic acid and Schiff’s solution (AB-PAS, pH 2.5) reaction.

### 2.5. Scanning Electron Microscopy

For scanning electron microscopy, specimens (*n* = 5) were prepared with modifications to the method described in [26]. The specimens were fixed for 4 h in 2.5% glutaraldehyde in 0.1 M phosphate buffer (pH 7.4), then rinsed three times for 20 min each in the same buffer. To remove organic material from the surface, the specimens were treated with 30% hydrogen peroxide for 24 h, dehydrated through a graded ethanol series from 50% to 100%, and dried. The specimens were subsequently platinum-coated at 10 mA for 4 min using a sputter coater (SC7620, Quorum Technologies, Laughton, UK) and examined with a field-emission scanning electron microscope (FE-SEM; Sigma 500, ZEISS, Oberkochen, Germany) at an accelerating voltage of 1 kV and scanning electron microscope (SEM; Axia ChemiSEM, Thermo Fisher Scientific, Waltham, WA, USA) at an accelerating voltage of 1~2 kV.

### 2.6. X-Ray Microscopy

For X-ray microscopy (XRM), a specimen (*n* = 1) was prepared with modifications to the method described by [27]. The Aristotle’s lantern was dissected from specimens fixed in 10% neutral buffered formalin, rinsed in running water for 48 h, and dehydrated through a graded ethanol series from 50% to 100%. The dehydrated specimens were embedded in Epon 812 and polymerized at 60 °C for 24 h. The embedded blocks were trimmed to approximately 15 × 15 × 11 mm and examined using an X-ray microscope (Xradia 620 Versa, ZEISS, Oberkochen, Germany) operated at 60 kV and 6.5 W. XRM imaging was performed with a voxel size of 5 μm using 3001 projections. Image acquisition and reconstruction were performed using the Scout-and-Scan Control System software 16.0 (ZEISS, Oberkochen, Germany).

### 2.7. Image Analysis

**Figure 3:**
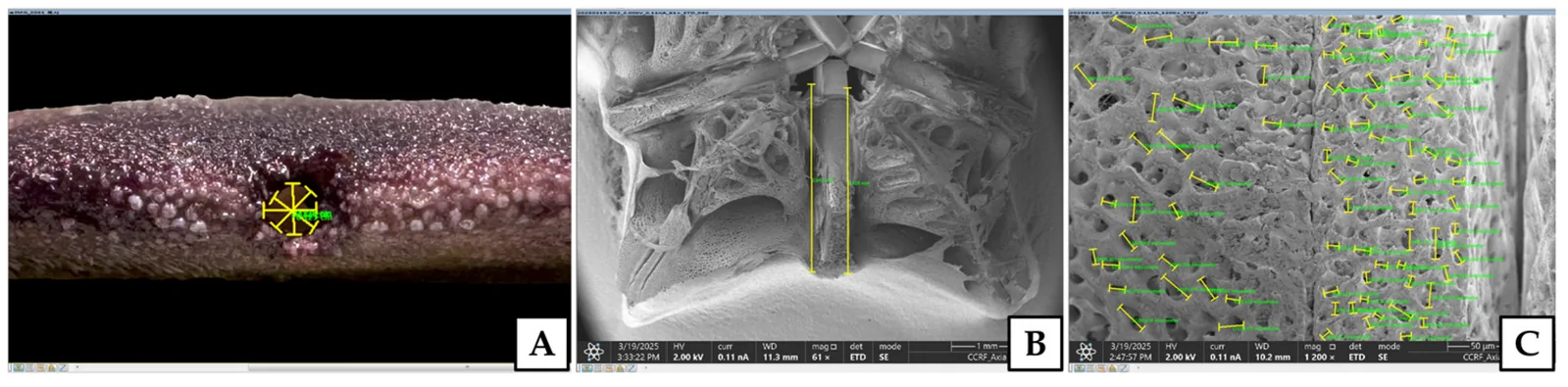
Image analysis of *Scaphechinus mirabilis*. (**A**): anus. (**B**): tooth slide. (**C**): demi-pyramid and epiphysis. Yellow lines indicate the measured lengths, and green labels indicate the corresponding measured values.

The major axis lengths of the mouth, anus, and ostium, the lengths of the pyramid and tooth, and the major axis lengths of the pores on the surfaces of the demi-pyramid, epiphysis, and rotula were measured using an image analyzer (i-Solution, IMT i-Solution Inc., Burnaby, Canada) (Figure 3).

### 2.8. Draw a Diagrammatic Representation

Diagrammatic representations were prepared using Procreate^®^ version 5.3.12 (Savage Interactive Pty Ltd., Hobart, Australia).

## 3. Results

### 3.1. External Morphology

**Figure 4:**
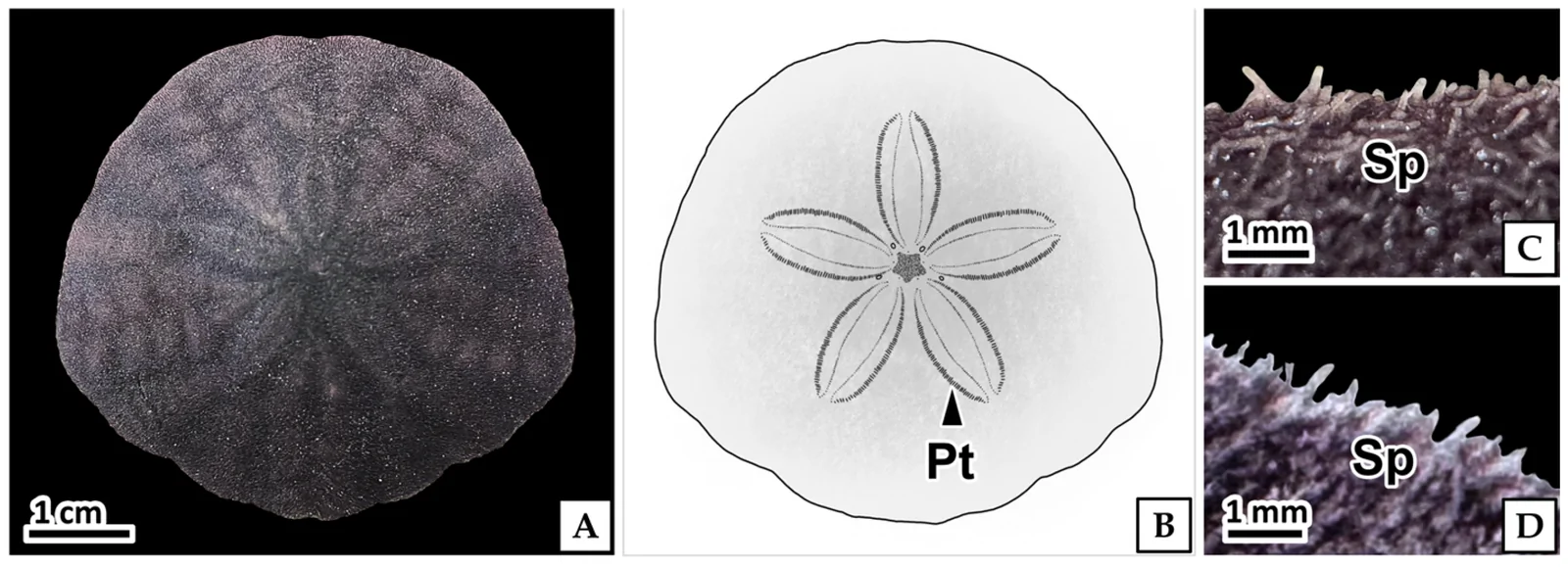
Morphology (**A**,**B**) of *Scaphechinus mirabilis*. (**A**): dorsal view. (**B**): schematic diagram of petaloids (Pt). (**C**,**D**): dorsal (**C**) and ventral (**D**) view. Sp: spine.

**Figure 5:**
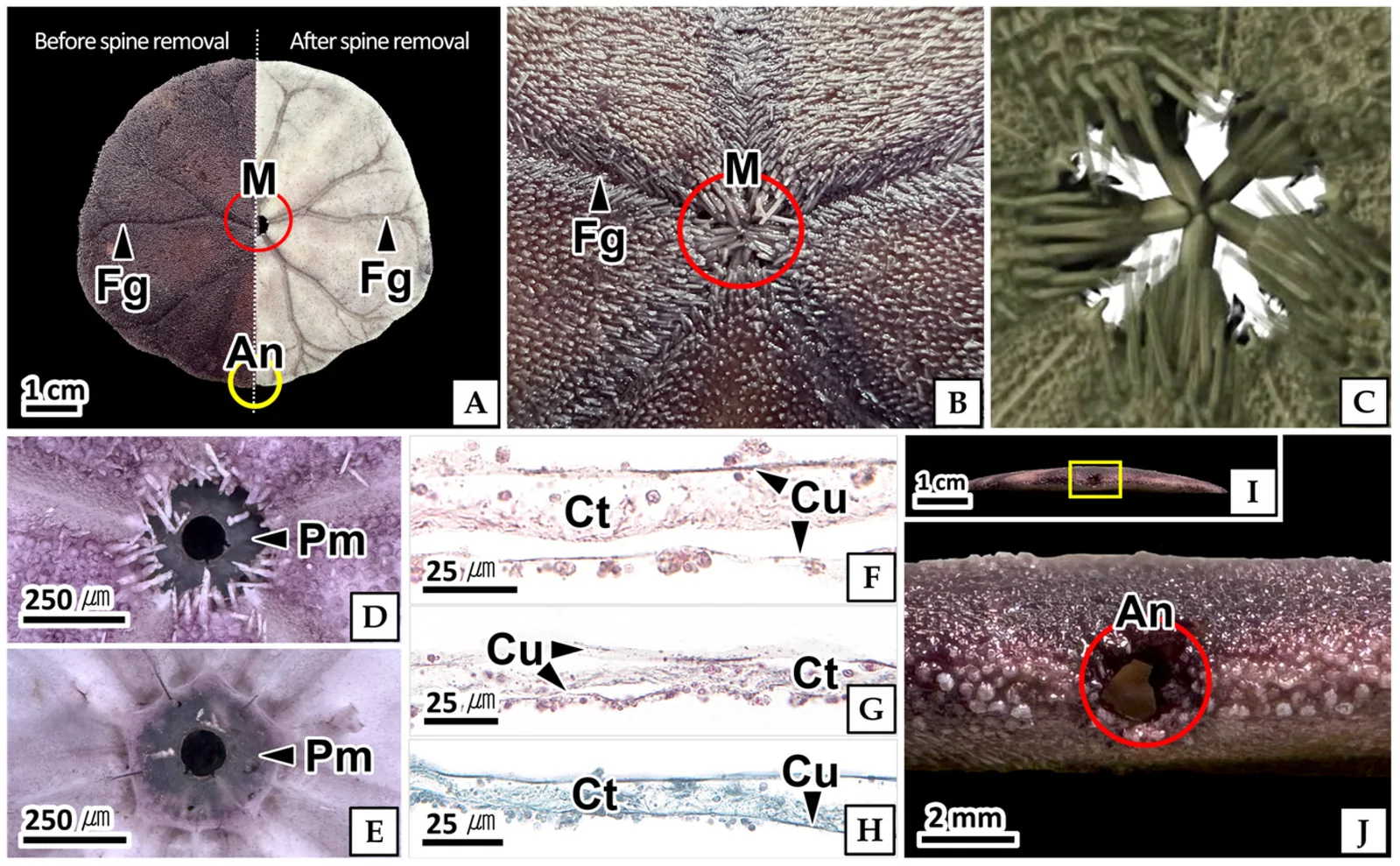
Mouth (M), peristomal membrane (Pm), and anus (An) of *Scaphechinus mirabilis*. (**A**–**C**): ventral view showing the mouth and food groove (Fg); (**C**): 3D XRM reconstruction of mouth. (**D**–**H**): peristomal membrane; (**D**,**E**): ventral (**D**) and dorsal (**E**) views showing the surface morphology; (**F**–**H**): light micrographs of cross sections showing the cuticle (Cu) and connective tissue (Ct). (**I**,**J**): lateral view. (**F**): H-E stain. (**G**): Masson’s trichrome stain. (**H**): AB-PAS (pH 2.5) reaction.

*Scaphechinus mirabilis* had a bilaterally symmetrical, rounded, and flattened disc-shaped test. The ventral surface was flat, whereas the dorsal surface formed a low dome (Figure 4A and Figure 5I). The external surface was covered with purple spines 1.01 (±0.18) mm in length (Figure 4C,D). Five petaloids were radially arranged on the dorsal surface (Figure 4A,B), whereas the food grooves and mouth were located on the ventral surface (Figure 5A).

The food grooves exhibited a root-like branching pattern. Multiple grooves originating from the test margin gradually converged into five main food grooves that extended to the mouth (Figure 5A,B).

The mouth was circular, 2.92 (±0.28) mm in diameter, and located at the center of the ventral surface (Figure 5B,C). It was covered by the peristomal membrane (Figure 5D,E). The peristomal membrane consisted of an outer cuticular layer and an inner connective tissue layer. The cuticular layer stained red with H-E stain and Masson’s trichrome stain and blue with AB-PAS (pH 2.5) reaction. The connective tissue layer consisted of dense connective tissue containing collagen fibers. It stained red with H-E stain and blue with Masson’s trichrome stain and AB-PAS (pH 2.5) reaction (Figure 5F–H).

The anus was circular, 1.35 (±0.07) mm in diameter, and located laterally along the horizontal body axis (Figure 5I,J).

### 3.2. Anatomical Structure

**Figure 6:**
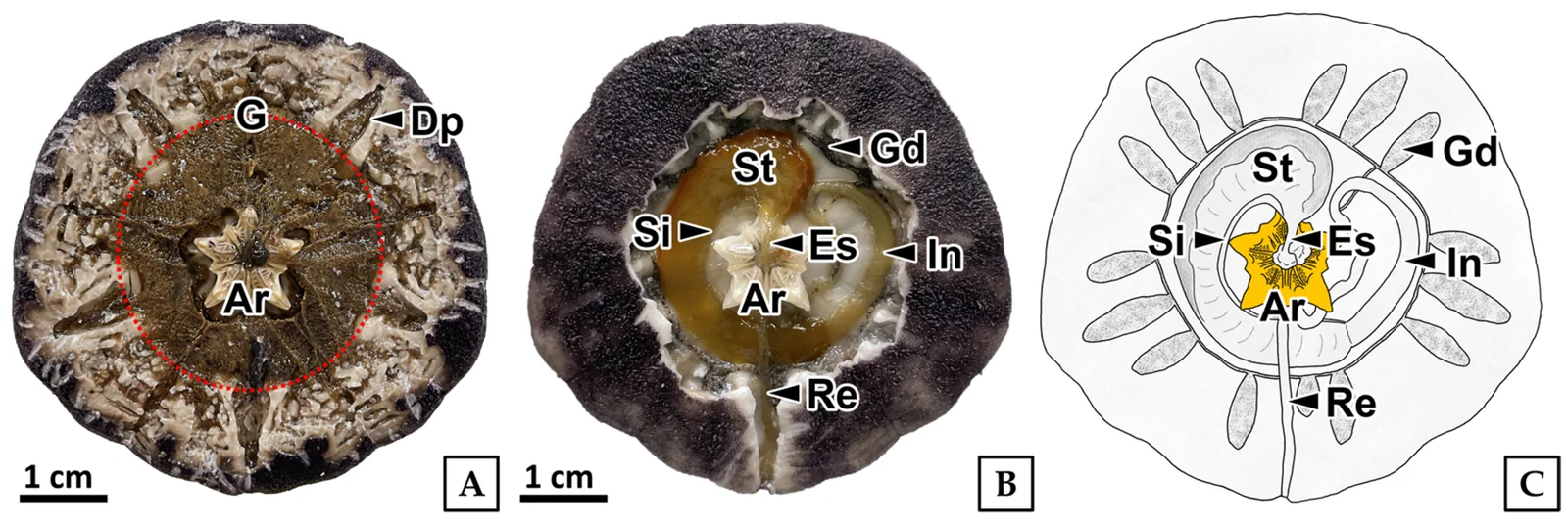
Anatomy (**A**,**B**) and schematic diagram (**C**) of digestive system in *Scaphechinus mirabilis*. A and B: dorsal view after removal of the test (**A**) and after subsequent removal of the gonad (**B**). The red line in (A) indicates the gonadal region. Ar: Aristotle’s lantern, Dp: diverticular pouch, Es: esophagus, G: gonad, Gd: Gregory’s diverticulum, In: intestine, Re: rectum, Si: siphon, St: stomach.

After removal of the dorsal test, the gonads, diverticular pouches, and Aristotle’s lantern were observed. The gonads were rounded, pentagonal, and brown to dark grayish-green, partially covering the Aristotle’s lantern and the digestive tract (Figure 6A). The diverticular pouches were located between the septa and contained Gregory’s diverticula (Figure 6A). The digestive system consisted of the pharynx, esophagus, stomach, siphon, intestine, rectum, and Gregory’s diverticulum (Figure 6B,C). The pharynx was located at the center of Aristotle’s lantern and extended from the mouth to the esophagus. The esophagus was a straight tube extending from the point where the pharynx exited Aristotle’s lantern to the stomach. At its distal end, it bifurcated into the stomach and siphon. The stomach was an expanded outer tube, whereas the siphon was a slender inner tube. Both the stomach and siphon merged into the intestine. The anterior intestine formed a loop, whereas the posterior rectum extended as a straight tube to the anus. Gregory’s diverticulum consisted of 15 pouches branching from the intestine (Figure 6B,C).

### 3.3. Aristotle’s Lantern

**Figure 7:**
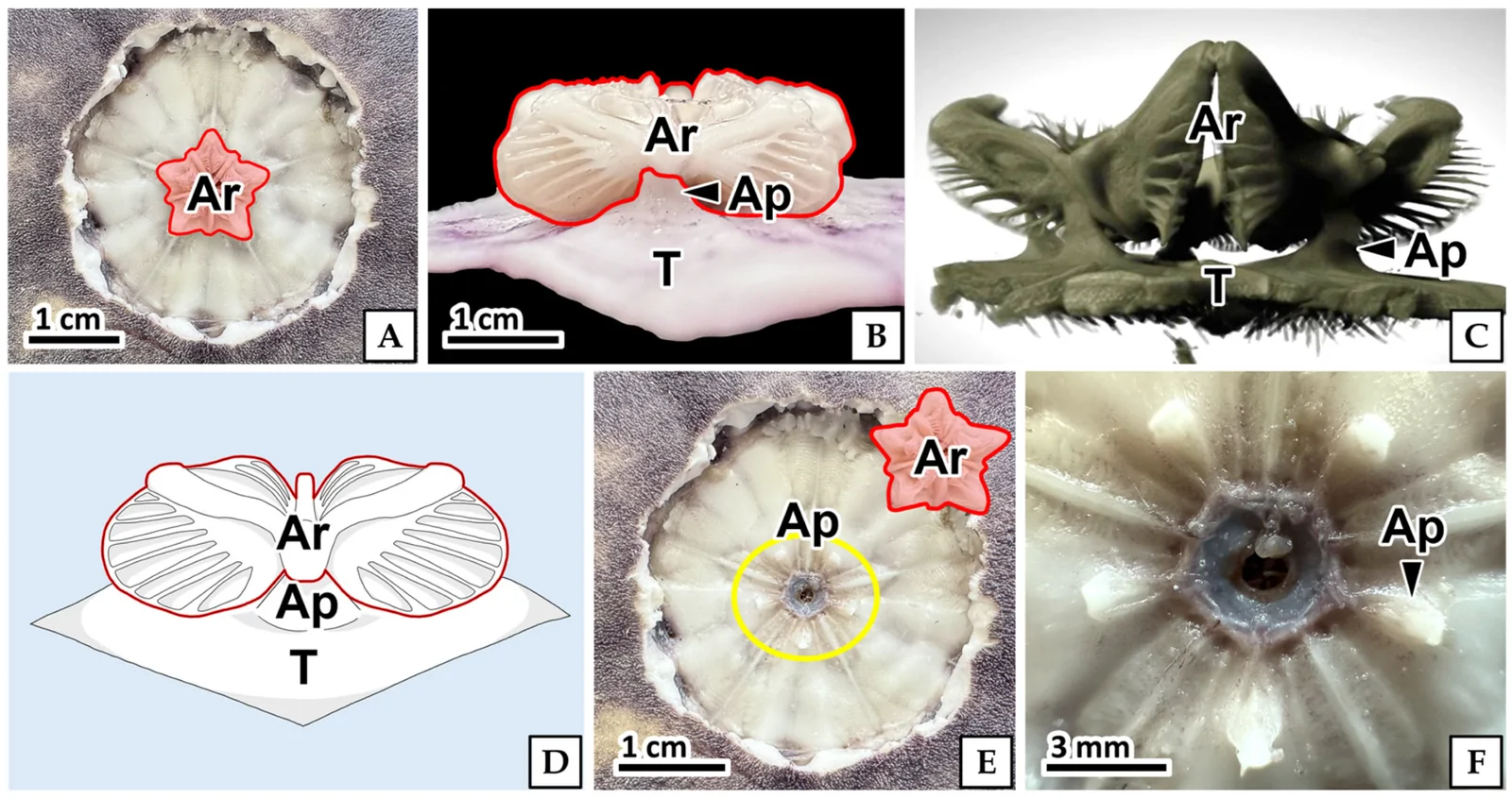
Aristotle’s lantern (Ar) on the apophysis (Ap) in *Scaphechinus mirabilis*. (**A**): dorsal view photograph after removal of the dorsal test (T). (**B**–**D**): lateral view showing the positional relationship between Aristotle’s lantern and the apophysis; (**B**): stereo micrographs; (**C**): 3D XRM reconstruction; (**D**): schematic diagram of (**B**). (**E**,**F**): apophysis after removal of the Aristotle’s lantern. Red outlines indicate Aristotle’s lantern, and the yellow circle in (**E**) indicates the apophysis.

**Figure 8:**
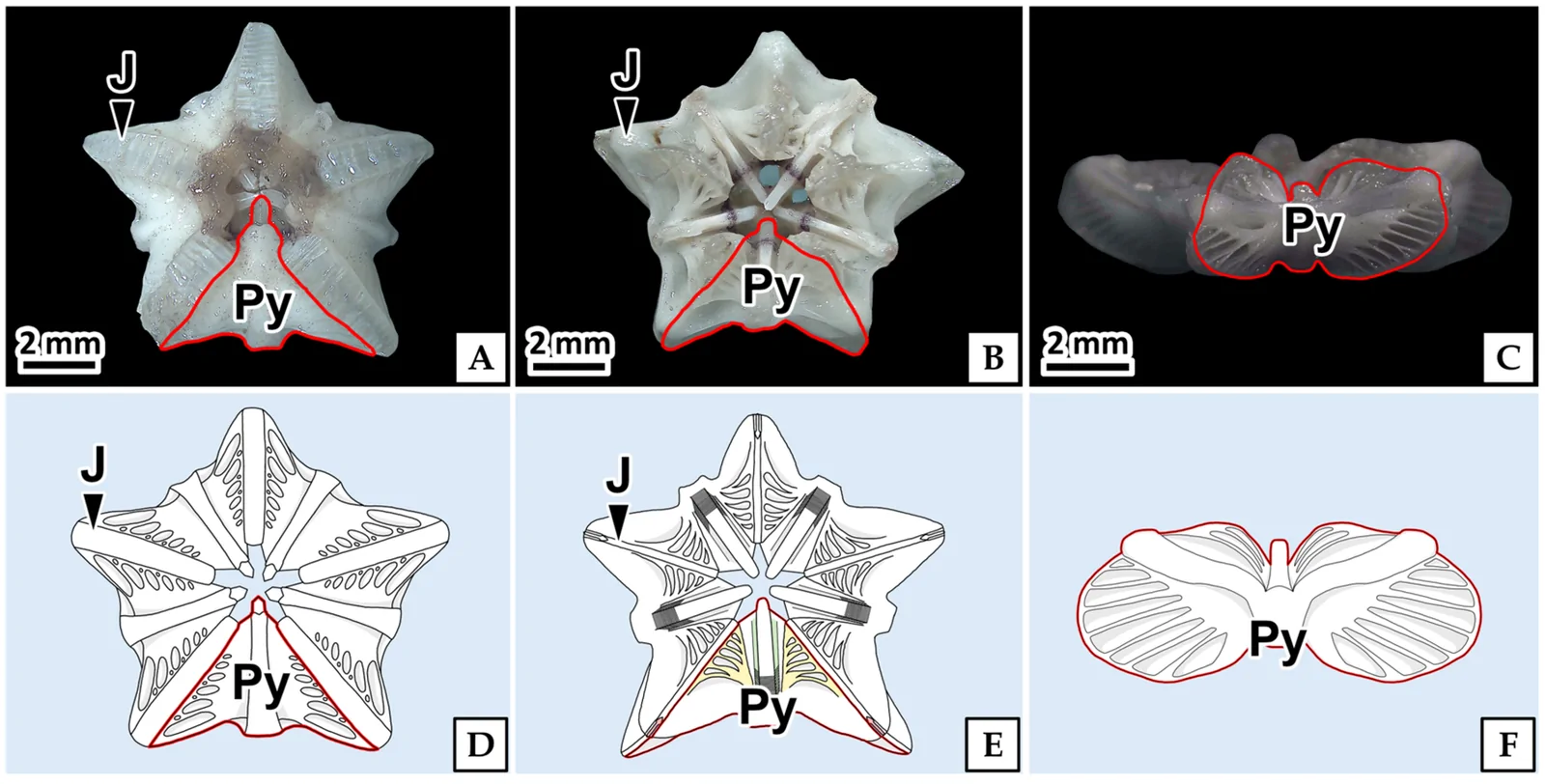
Aristotle’s lantern in *Scaphechinus mirabilis*. (**A**–**C**): photographs showing the five pyramids (Py, red line) connected by joints (J). (**D**–**F**): schematic diagram of (**A**–**C**). (**A**,**D**): ventral view. (**B**,**E**): dorsal view. (**C**,**F**): lateral view.

Aristotle’s lantern was attached to the test by five apophyses (Figure 7A–D). The apophyses were trapezoidal projections arising from the inner surface of the test and possessed grooves that supported Aristotle’s lantern (Figure 7E,F). Aristotle’s lantern was a milky-white, star-shaped structure measuring 9.96 (±0.59) mm in length and consisted of five pyramids connected by joints (Figure 8).

#### 3.3.1. Pyramid

**Figure 9:**
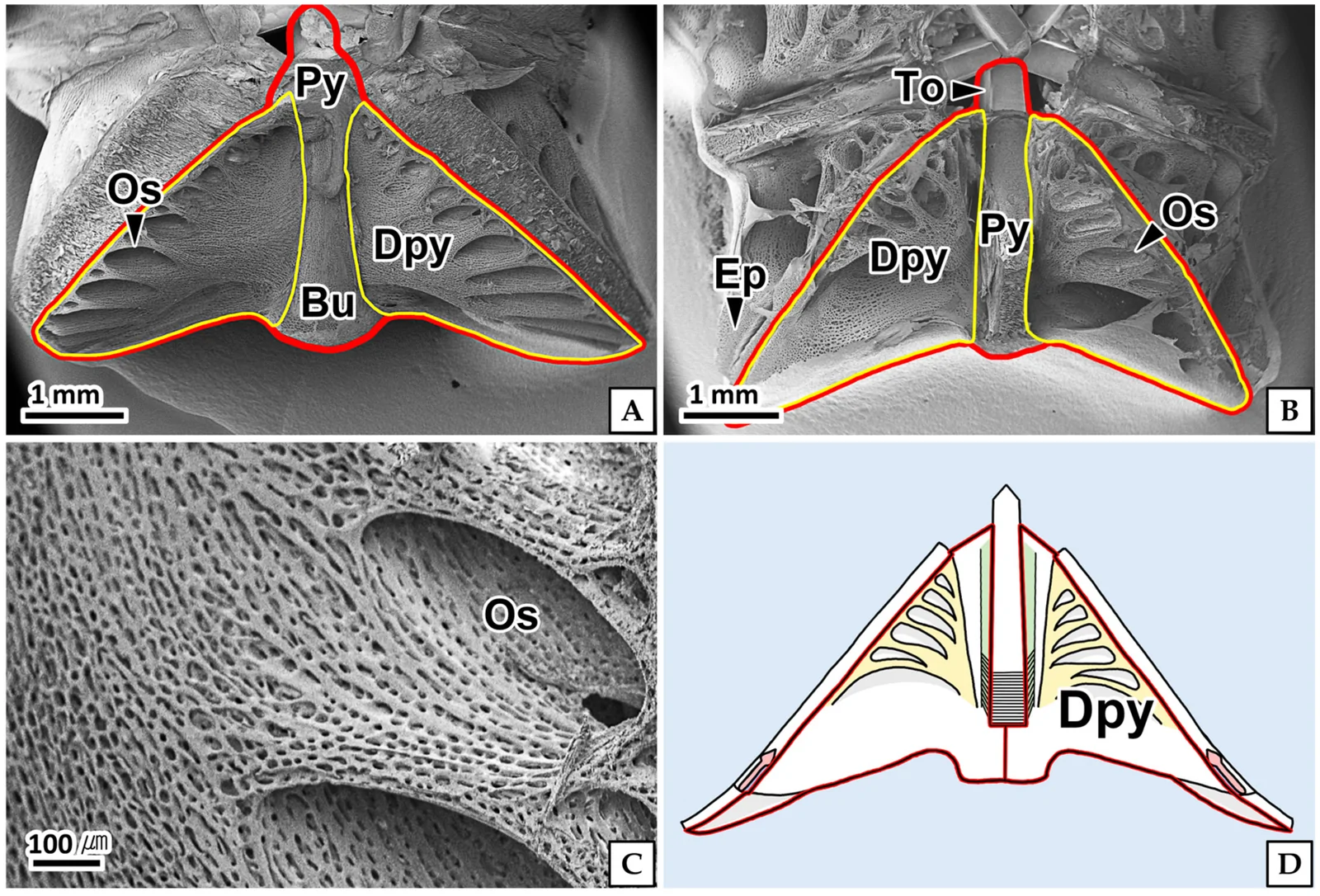
Pyramid (Py, red outline) of Aristotle’s lantern in *Scaphechinus mirabilis*. The pyramid is composed of two demi-pyramids (Dpy, yellow outline). (**A**–**C**): Scanning electron micrographs; (**A**): ventral view; (**B**): dorsal view; (**C**): ostium (Os) on the demi-pyramid. (**D**): schematic diagram of (**B**). Bu: bump, Ep: epiphysis, To: tooth.

The pyramid was butterfly-shaped and consisted of two demi-pyramids (Figure 9A,B). The ventral surface of the pyramid bore a protruding bump (Figure 9A), whereas the dorsal surface bore the tooth and epiphysis (Figure 9B).

Each demi-pyramid was triangular and exhibited a porous structure with 5–7 depressed oval ostia surrounding the raised area. The major axis length of the ostium differed between regions, measuring 0.40 (±0.08) mm on the dorsal side and 0.65 (±0.35) mm on the ventral side (Figure 9A–C). Each ostium was an independent structure with no interconnections between adjacent ostia (Figure 9C).

#### 3.3.2. Joint

**Figure 10:**
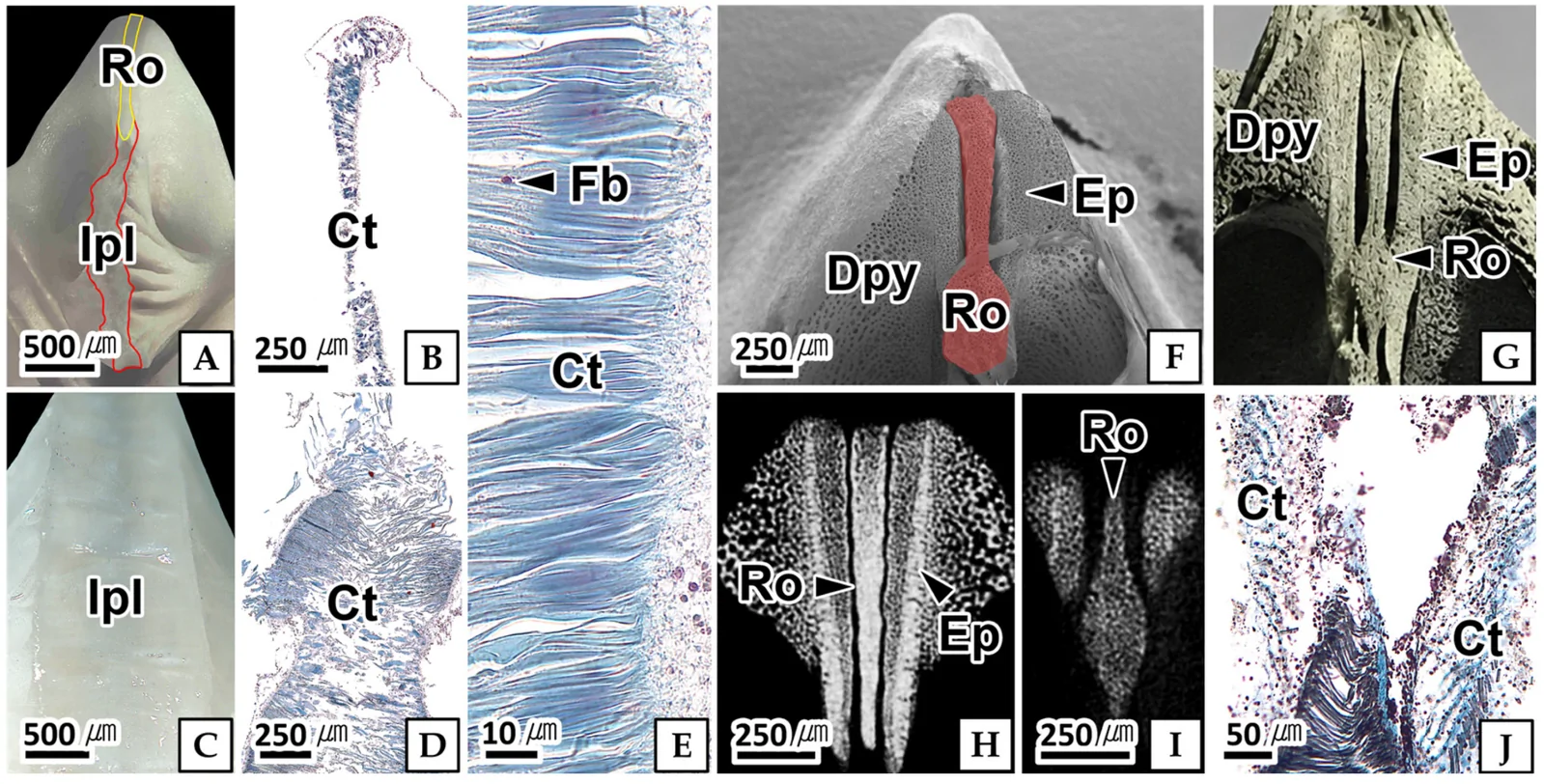
Joint of Aristotle’s lantern in *Scaphechinus mirabilis*. The joint consists of the interpyramidal ligament (Ipl, red outline) and rotula (Ro). (**A**,**C**): photographs of the joint. (**B**,**D**,**E**): light micrographs of the interpyramidal ligament; (**E**): connective tissue (Ct) and fibroblast (Fb). (**F**): scanning electron micrographs of the rotula (red colored area). (**G**): 3D XRM reconstruction. (**H**,**I**): sequential virtual 2D XRM sections showing changes in the shape of the rotula. (**J**): connective tissue connecting the rotula to the adjacent epiphyses (Ep). (**A**,**B**,**F**–**J**): dorsal view, (**C**–**E**): ventral view. Dpy: demi-pyramid. Masson’s trichrome stain.

The joint was located between adjacent pyramids and consisted of the interpyramidal ligament and rotula (Figure 10A,B).

The interpyramidal ligament differed in length between regions, measuring 0.09 (±0.02) mm on the dorsal side and 0.64 (±0.18) mm on the ventral side (Figure 10A–D). It consisted of dense connective tissue composed of collagen fibers and fibroblasts and stained blue with Masson’s trichrome stain. Fibroblasts were scattered among the collagen fibers, and their nuclei occupied most of the cell, appearing purple with Masson’s trichrome stain (Figure 10C–E).

The rotula was located dorsal to the interpyramidal ligament (Figure 10A). It was spoon-shaped with a porous structure and measured 1.37 (±0.06) mm in height. The cylindrical stalk measured 0.13 (±0.02) × 0.82 (±0.03) mm and was positioned between the paired epiphyses, whereas the hexagonal base measured 0.32 (±0.01) × 0.62 (±0.02) mm and was located on the outer dorsal side of the paired epiphyses (Figure 10F,G). Serial X-ray microscopy sections showed that the cross-sectional area of the rotula gradually increased from the ventral to the dorsal side (Figure 10H,I). The rotula was connected to the adjacent epiphyses by connective tissue located above the interpyramidal ligament. The connective tissue consisted of collagen fibers (Figure 10J).

#### 3.3.3. Epiphysis

**Figure 11:**
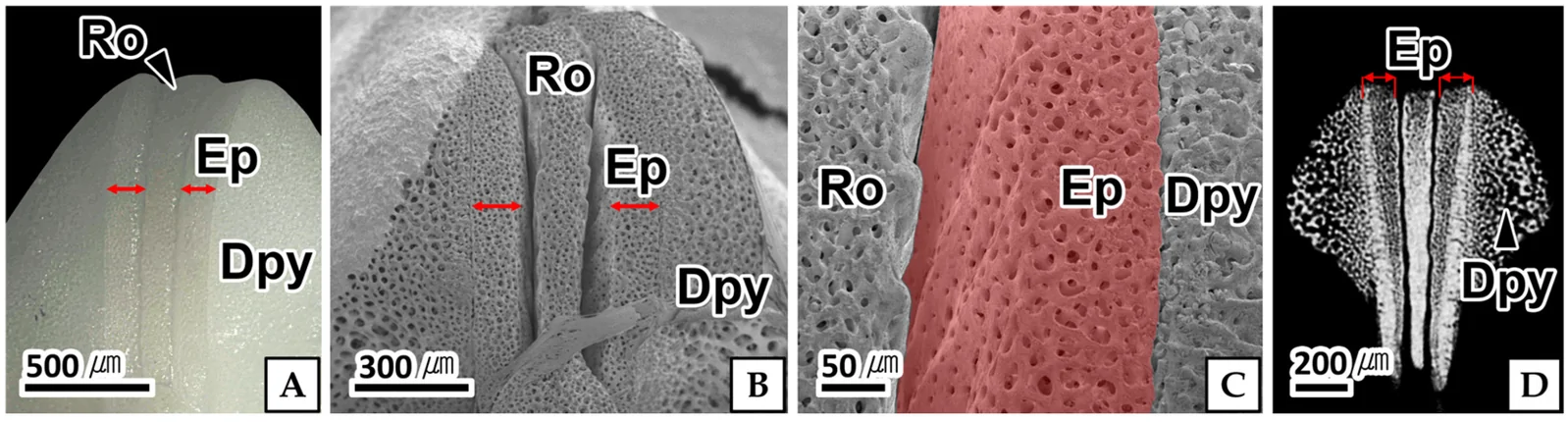
Epiphysis (Ep) of Aristotle’s lantern in *Scaphechinus mirabilis*. Red arrows and red-colored area indicate the region of the epiphysis. The epiphysis is located between the demi-pyramids (Dpy) and the rotula (Ro). (**A**): photograph of the epiphysis. (**B**,**C**): scanning electron micrographs. (**D**): virtual 2D XRM section showing the relatively higher X-ray signal intensity of the epiphysis than the adjacent demi-pyramids.

The epiphysis was located between the demi-pyramid and the rotula (Figure 11A–C).

The epiphysis was cuboidal, 0.91 (±0.08) mm in length, and had an inclined surface facing the rotula (Figure 11A). The epiphysis exhibited a porous structure (Figure 11B,C), and the surface pores measured 0.01 mm in diameter. In X-ray microscopy, the epiphysis exhibited relatively higher X-ray signal intensity than the adjacent demi-pyramid (Figure 11D).

#### 3.3.4. Tooth

**Figure 12:**
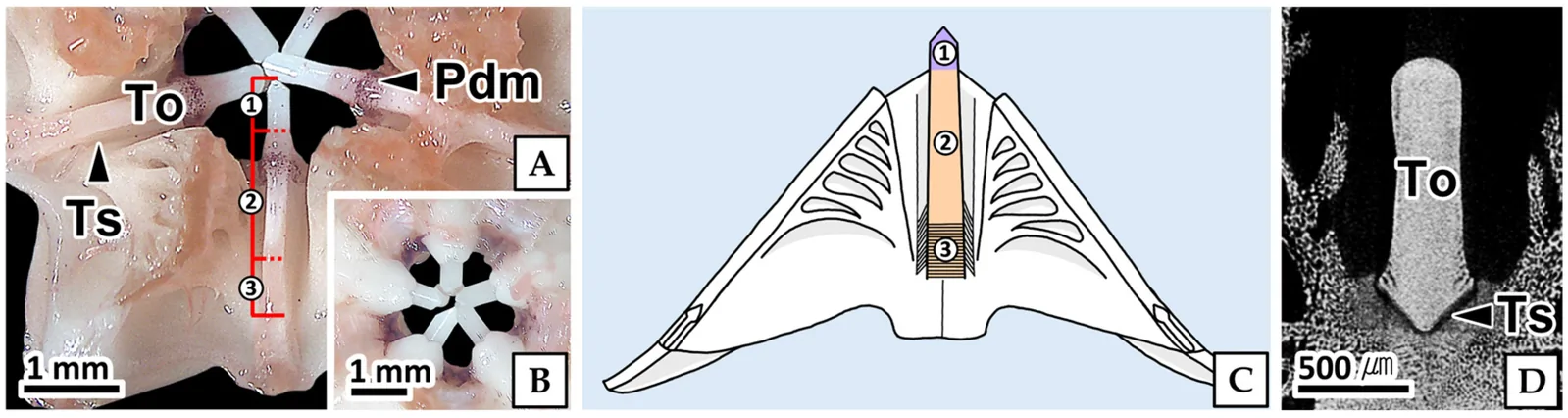
Tooth (To) of Aristotle’s lantern in *Scaphechinus mirabilis*. (**A**,**B**): photographs of dorsal (**A**) and ventral (**B**) views. (**C**): schematic diagram of (**A**). (**D**): virtual 2D XRM section showing the tooth (To) within the tooth slide (Ts). Red lines indicate the regions of the three parts of the tooth. ① apical projection part; ② basal projection part covered by the peridental membrane (Pdm); ③ embedded part.

The tooth attached to Aristotle’s lantern was divided into an embedded part and a projection part (Figure 12A–C). The embedded part was located within the tooth slide of the pyramid and was connected to the dental ligament and dental promoter muscle (Figure 12A,D). It accounted for approximately 15% of the total tooth length. The projection part accounted for approximately 85% of the total tooth length and was divided into a basal projection part, which was located within the tooth slide and covered by the peridental membrane, and an apical projection part, which projected beyond the tooth slide (Figure 12A–C).

**Figure 13:**
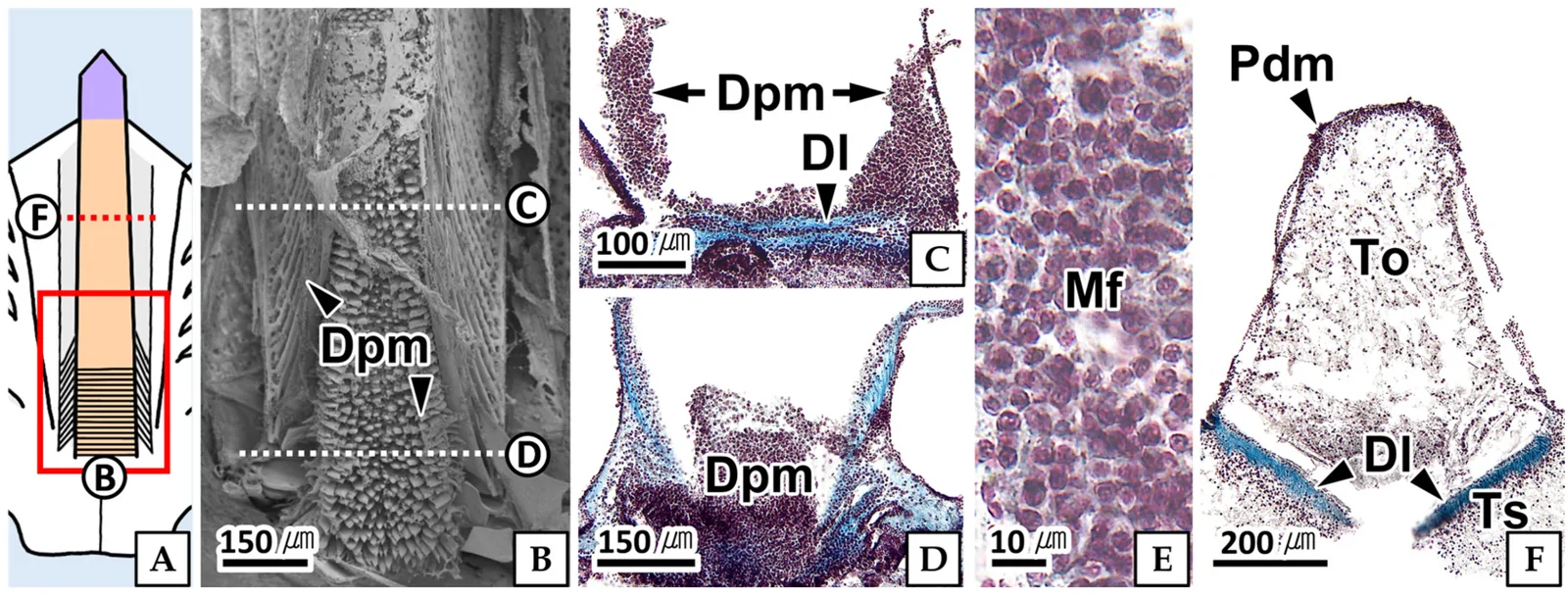
Dental ligament (Dl) and dental promoter muscle (Dpm) of the pyramid in *Scaphechinus mirabilis*. (**A**): schematic diagram of the tooth (To). The red rectangle indicates the region shown in (**B**), and the red line indicates the sectioning site shown in (**F**). (**B**): scanning electron micrograph of embedded part. The white line indicates the sectioning site shown in (**C,D**). (**C**–**F**): light micrographs; (**C**,**D**): dental promoter muscle located on the lateral (**C**) and posterior (**D**) side of the tooth. (**E**): muscle fiber (Mf) of dental promoter muscle. (**F**): tooth covered by the peridental membrane (Pdm) and attached to the tooth slide (Ts) by the dental ligament. Masson’s trichrome stain.

The embedded part was surrounded by a well-developed muscular layer composed of smooth muscle and connective tissue. Around the tooth, the inner layer consisted of circular smooth muscle, whereas the outer layer consisted of longitudinal smooth muscle (Figure 13B–E).

The tooth slide was a V-shaped groove, 2.14 (±0.03) mm in length and 0.40 (±0.04) mm in width, located at the center of the dorsal side of the pyramid. The tooth was connected to the tooth slide by the dental ligament (Figure 12D and Figure 13D).

The peridental membrane was a thin connective tissue layer, 12.92 (±4.50) μm in thickness, connecting to the tooth slide (Figure 13D). The dental ligament was located on the surface of the tooth slide. It was straight along the embedded part (Figure 13C,D) but formed a V-shaped configuration near the projection part, following the contour of the tooth surface (Figure 13F)

**Figure 14:**
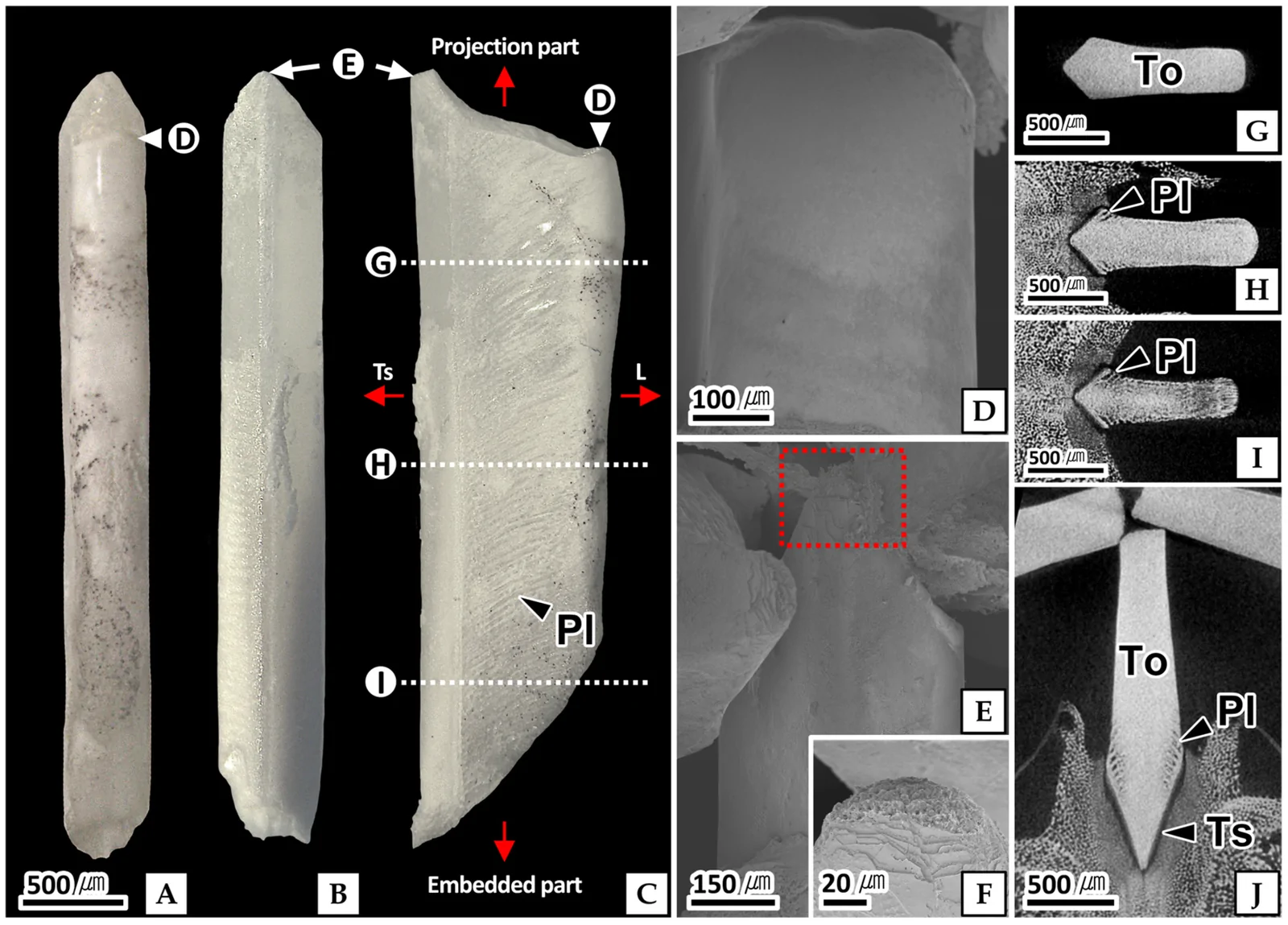
Isolated tooth (To) from Aristotle’s lantern of *Scaphechinus mirabilis*. (**A**–**C**): photographs of tooth. The white arrows indicated the regions shown in (**D**) and (**E**), and the red arrows indicate the orientation of the tooth. (**D**–**F**): Scanning electron micrographs showing the multilayered structure of the projection part. The red box in (**E**) indicates the region shown in (**F**). (**G**–**J**): 3D XRM images; (**G**–**I**): sequential virtual 2D XRM sections corresponding to the regions indicated by the white line in (**C**), showing the plumula (Pl) from the projection part toward the embedded part of the tooth; (**J**): plumula at the basal region of the tooth. (**A**,**D**): dorsal view. (**B**,**E**,**F**): ventral view. (**C**): lateral view. L: lumen, Ts: tooth slide.

The tooth isolated from Aristotle’s lantern was milky white and elongated trapezoidal, measuring 3.35 (±0.35) mm in length, 0.98 (±0.05) mm in width, and 0.38 (±0.01) mm in thickness (Figure 14A–C). The apex of the apical projection part was rounded on the dorsal side (Figure 14D) and pointed on the ventral side (Figure 14E). In cross section, the tooth exhibited 15–20 concentric lamellae, each approximately 0.76 (±0.18) μm thick. The ventral surface of the apical projection part exhibited wean (Figure 14F). In X-ray microscopy, multiple calyx-shaped plumula extended from the basal projection part to the embedded part and became progressively larger toward the embedded part (Figure 14G–J).

#### 3.3.5. Bump

**Figure 15:**
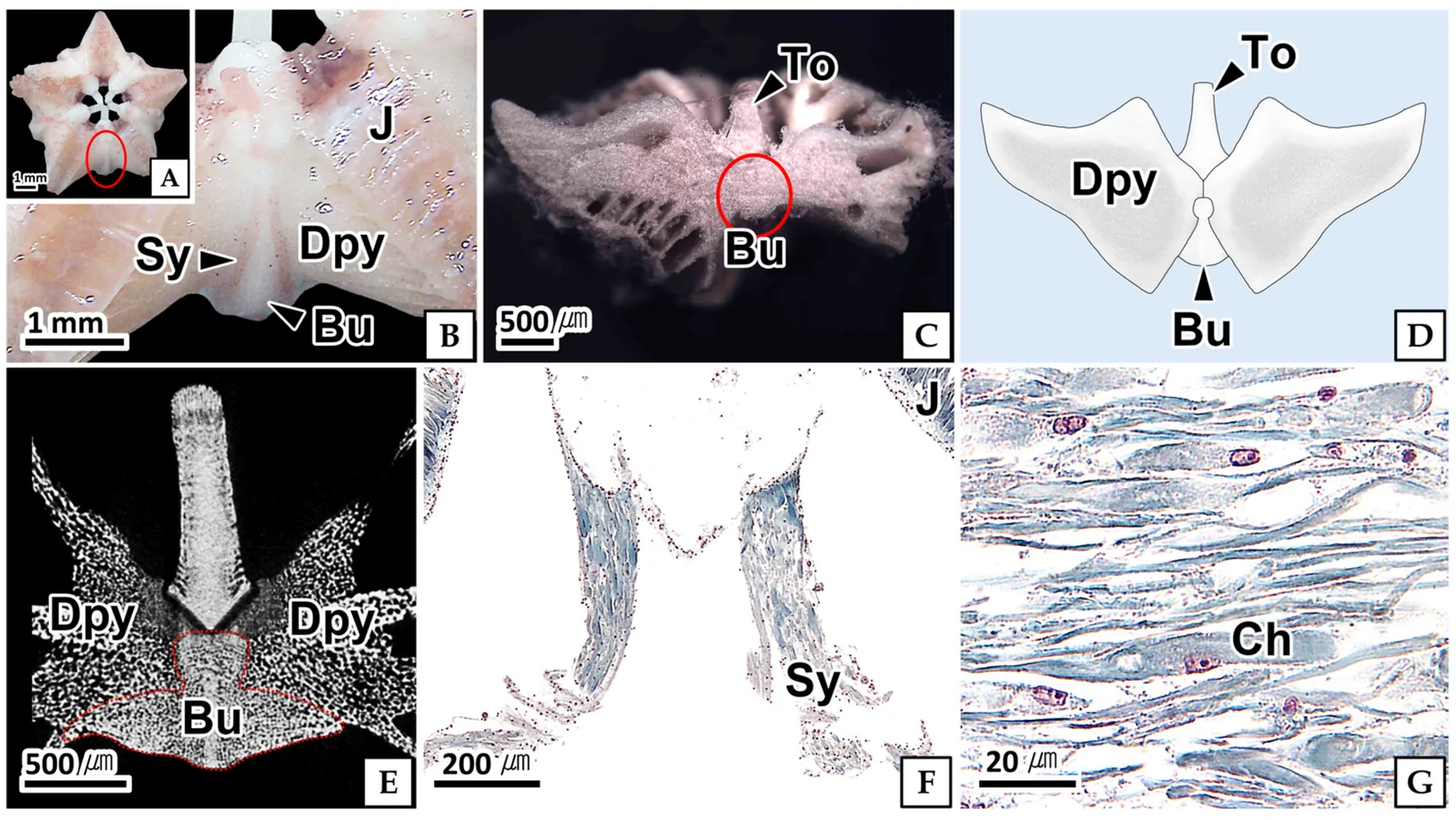
Bump (Bu) of Aristotle’s lantern in *Scaphechinus mirabilis*. Red circles and outline indicate the region of the bump. (**A**,**B**): ventral views; (**B**): symphysis (Sy) connecting the bump to the paired demi-pyramids (Dpy). (**C**–**E**): lateral views; (**C**): ground specimen; (**D**): schematic diagram of (**C**); (**E**): virtual 2D section from the XRM dataset showing the bump with higher X-ray signal intensity than the adjacent demi-pyramids. (**F**,**G**): light micrographs of longitudinal sections of the symphysis; (**F**): fibrous cartilage composed of dense connective tissue; (**G**): fibrous chondrocytes (Ch). To: tooth. Masson’s trichrome stain.

The bump was located at the lower part of the junction between the two demi-pyramids on the ventral side. The junction between the demi-pyramids was covered by the symphysis (Figure 15A,B). The bump was a semi-cylindrical column with a fan-shaped base, measuring approximately 0.5 mm in height (Figure 15B). In the cross section of the ground specimen, a pale line separating the bump from the adjacent demi-pyramid was observed (Figure 15C). The bump was readily distinguished from the adjacent demi-pyramid in the X-ray images, showing a more homogeneous internal structure (Figure 15E). The symphysis was stained light pink with 0.5% eosin (Figure 15B). Histologically, the tissue consisted of chondrocytes and a collagen-rich extracellular matrix. In Masson’s trichrome-stained sections, the chondrocytes were stained red, whereas the collagen-rich extracellular matrix was stained blue (Figure 15F,G).

## 4. Discussion

Echinoids differ among taxonomic groups in habitat and feeding methods. As a result, their external morphology, digestive tract morphology and organization, and the morphology of Aristotle’s lantern, the feeding apparatus, differ accordingly [28].

Irregular echinoids possess short aboral spines and food grooves that transport food particles toward the mouth through the action of oral spines and tube feet [29,30]. Similar feeding structures have been described in other clypeasteroids, including *Mellita quinqujesperforata* [29], *Dendraster excentricus* [31], and *Astriclypeus manni* [11]. Likewise, *Scaphechinus mirabilis* possesses short aboral spines (≤5 mm) and five petaloid food grooves extending to the mouth. These morphological features are consistent with the transport of food particles toward the mouth described for other clypeasteroids.

The mouth of echinoids is surrounded by the peristomal membrane. The peristomal membrane is a disc-shaped membranous structure connecting the test and Aristotle’s lantern [32]. It functions as a diaphragm-like viscoelastic ligament that actively or passively participates in all movements of the lantern [33].

In regular echinoids, the peristomal membrane consists of a dermis containing mutable collagenous tissue, a transitional epithelium, and a basiepithelial nerve plexus [33]. In contrast, Cidaroids possess skeletal plates extending into the peristomal membrane and lack mutable collagenous tissue, resulting in reduced dynamic function [34]. In addition, the Aristotle’s lantern of irregular echinoids in the order Clypeasteroida is flattened, non-protrusible, and adapted for crushing sediment during feeding [35]. The peristomal membrane of *S. mirabilis* was disc-shaped and attached to the mouth, consisting of an outer cuticle layer and an inner connective tissue layer. This histological organization differed from that reported in regular echinoids and may be associated with supporting the tissues surrounding the mouth and maintaining the connection with Aristotle’s lantern.

The Aristotle’s lantern is the feeding apparatus of echinoids, and its morphology and degree of development vary among species. Regular echinoids possess an Aristotle’s lantern with a wide range of tooth movement for grazing on food such as algae, and larger lanterns have been reported to increase feeding efficiency [17]. The morphology of Aristotle’s lantern in regular echinoids varies among taxonomic groups. The major anatomical characteristics of Aristotle’s lantern in regular echinoids, irregular echinoids, and *S. mirabilis* are summarized in Table 2. In echinoids belonging to the orders Cidaroida, Diadematacea, Arbacioida, and Camarodonta, the Aristotle’s lantern is lantern-shaped [36]. In irregular echinoids, the morphology of Aristotle’s lantern has been described for Cassiduloida, Oligopygoida, Clypeasterina, Laganina, and Scutellina [12]. In Cassiduloida, Aristotle’s lantern is present only during the juvenile stage and has an upright morphology similar to that of regular echinoids. Oligopygoida and Clypeasterina possess a prominent supra-alveolar process located at the center of the lantern. In contrast, in Laganina and Scutellina, the supra-alveolar process is located at the lateral margin of the lantern. In Laganina, the rotula is greatly reduced or absent in some taxa, whereas Scutellina possesses a long and narrow rotula located between the adjacent epiphyses [12]. In the present study, *S. mirabilis* was similar to Scutellina in having a cylindrical stalk of the rotula located between the epiphyses. In contrast, the hexagonal base of the rotula was located on the outer upper margin of the epiphyses rather than between them, differing from that of Scutellina.

In regular echinoids, Aristotle’s lantern is broadly divided into the skeletal system and the muscular and ligamentous systems. The skeletal system and the muscular and ligamentous systems interact to produce various movements, including protraction, retraction, and lateral tilting of the overall apparatus, and opening and closing of the five jaws [37]. The skeletal system consists of the pyramids, teeth, epiphyses, rotula, and compass [38], whereas the muscular and ligamentous systems consist of muscles and ligaments [37]. In contrast, the Aristotle’s lantern of *S. mirabilis* lacked a compass in the skeletal system, whereas the muscular and ligamentous systems consisted of the interpyramidal ligament, dental ligament, and dental promoter muscle, differing from those of regular echinoids.

The compass is located above the rotula and undergoes vertical movements independent of the lantern. It has been suggested to be involved in the movement and circulation of coelomic fluid within the peripharyngeal coelom surrounding the lantern [37]. In contrast, *S. mirabilis* lacks a compass, and further studies are needed to clarify the mechanism of coelomic fluid circulation.

In *S. mirabilis*, all skeletal components except the teeth exhibited a porous structure, consistent with the Aristotle’s lantern of the regular echinoids *Stylocidaris affinis* [37] and *Diadema setosum* [20].

In both regular and irregular echinoids, each pyramid consists of two demi-pyramids [16]. In regular echinoids, the demi-pyramids are flattened structures with depressions for muscle attachment and lack ostium on their surfaces [37]. In contrast, in *Fellaster zelandiae* of Echinolampadacea, ostium is located adjacent to the joint and is interconnected by a stereom structure [39]. In the present study, the pyramid of *S. mirabilis* consisted of two demi-pyramids, each with a raised area. Both the dorsal and ventral sides of the demi-pyramids possessed 5–7 oval ostia adjacent to the joint. However, unlike those of *F. zelandiae*, each ostium was an independent structure lacking interconnections [39].

In regular echinoids, the joints of Aristotle’s lantern are divided into the perignathic girdle–lantern joint and the rotular joint. The perignathic girdle–lantern joint connects the lantern to the test through muscles and ligaments and is responsible for protraction, retraction, and lateral tilting of the lantern, whereas the rotular joint connects adjacent pyramids and is responsible for opening and closing movements of the lantern [37].

The rotular joint consists of the rotula and ligaments and is classified into socket joints and hinge joints according to the position of the rotula. In the socket joint, the rotula is located medial to the epiphysis and is mainly found in Cidaroids, whereas in the hinge joint, the rotula is positioned at the same level as the epiphysis and is characteristic of echinoids [16]. In the present study, unlike in regular echinoids, only the rotular joint was present in *S. mirabilis*, whereas the perignathic girdle–lantern joint was absent. In addition, the rotular joint consisted of the rotula and the interpyramidal ligament, which was composed of connective tissue, and was classified as a hinge joint because the rotula was positioned at the same level as the epiphysis. Unlike that of regular echinoids, the Aristotle’s lantern of Echinolampadacea does not protrude beyond the mouth, and the teeth move horizontally against the sediment to crush it [13,21,29]. Based on this morphological arrangement, the teeth of *S. mirabilis* may move anteroposteriorly along the body axis.

In regular echinoids, the epiphysis is located at the upper part of the pyramid, where it provides an articulation site for the rotula and forms the joint. In *Eucidaris tribuloides*, the epiphysis has been reported to measure 2.5–3.0 mm in length [16]. In the present study, the epiphysis of *S. mirabilis* was smaller than that reported for *E. tribuloides.* In addition, the pores on the surface of the epiphysis were smaller than those on the surface of the demi-pyramid, and X-ray microscopy revealed higher signal intensity in the epiphysis than in the adjacent demi-pyramid. The higher X-ray signal intensity and smaller surface pores in the epiphysis of *S. mirabilis* compared with the adjacent demi-pyramid suggest a denser stereom microstructure, consistent with the occurrence of denser stereom in echinoid skeletal regions subjected to greater mechanical stress [13,40]. Therefore, the relatively dense stereom of the epiphysis may be associated with the mechanical demands of the epiphysis–rotula articulation, although further biomechanical studies are required to confirm this functional relationship [13,41].

In regular echinoids, the teeth of Aristotle’s lantern are the primary structures responsible for scraping food and are U- or T-shaped columns. T-shaped teeth possess a well-developed keel that reinforces the tooth structure and are arranged perpendicular to the body axis [36,37,42]. In contrast, in Echinolampadacea, including *M. tenuis*, the teeth are arranged parallel to the body axis and are therefore unsuitable for scraping food. Instead, they function by crushing ingested sediment particles [21,29,43]. In the present study, the teeth of *S. mirabilis* were elongated trapezoidal structures, arranged parallel to the body axis on the tooth slide of the pyramid and anchored by the dental ligament. This arrangement was consistent with that reported for Echinolampadacea.

In regular echinoids, the teeth are anchored to the center of the pyramid by the dental ligament and move together with the pyramid through the action of the protractor and retractor muscles, which pull the pyramid upward and downward, respectively [44]. In *Echinarachnius parma* of Echinolampadacea, the teeth are located on the dorsal side of the pyramid, and a well-developed dental promoter muscle located at the posteroventral part of the tooth is responsible for tooth movement [15]. In the present study, muscles were well developed on both sides and at the base of the embedded part of the tooth in *S. mirabilis*. Similar to *E. parma* [15], the lateral muscles in *S. mirabilis* may be involved in the protraction and retraction of the tooth, whereas the circularly arranged muscles at the base may contribute to tooth stabilization.

In the regular echinoid *Strongylocentrotus purpuratus* [45] and the Echinolampadacea *E. parma* [15], the teeth possess a worn apical surface and a plumula at the basal region. The worn surface is formed as the teeth crush sediment particles, whereas the plumula indicates continuous tooth formation and growth from the basal region [15,18]. In the present study, *S. mirabilis* also exhibited a similar worn apical surface and a multilayered, calyx-shaped plumula. These findings suggest that the plumula is associated with continuous tooth formation and growth from the basal region and may also provide a structural basis for the firm attachment of the dental ligament and dental promoter muscle.

**Table 2:** Comparison of the major anatomical characteristics of Aristotle’s lantern in regular echinoids, irregular echinoids, and *Scaphechinus mirabilis.*

Feature	Regular Echinoids	Irregular Echinoids	Present Study
Lantern			
Shape	Lantern-shaped [15]	Flattened [15]	Flattened
Projection	Protrusible [15]	Non-protrusible [46]	Non-protrusible
Compass	Present [37]	Absent [12]	Absent
Rotula			
Position	Between epiphyses [46]	Between epiphyses [12]	Between epiphyses
Shape	Generally, not reduced [46]	Very small plate-like [12,46]	Cylindrical stalk
Pyramid	Composed of two demi-pyramids	Composed of two demi-pyramids	Composed of two demi-pyramids
Demi-pyramid	Flattened; Ostia absent [40]	Interconnected ostia adjacent to the joint [39]	5–7 independent ostia adjacent to the joint
Joint			
Perignathic girdle–lantern joint	Present [37]	-	Absent
Rotular joint	Socket or hinge type [16]	Flat planar surface [46]	Hinge type
Epiphysis	Upper part of pyramid [16]	On the demi-pyramids; Flanking the rotula [12]	Upper part of pyramid; Flanking the rotula
Tooth			
Arrangement	Perpendicular to body axis [36,37,42]	Parallel to body axis [21,29,43]	Parallel to body axis
Position		Located on the tooth slide [21,29,43]	Located on the tooth slide
Shape	U- or T-shape; Keeled [36,37,42]	Taxon-dependent	Elongated trapezoidal; Keel absent
Plumula structure	Present [45]	Present [15]	Present; Multilayered and calyx-shaped
Tooth movement	Moves with the pyramid [44]	Independent of pyramid movement [15]	May move independently of the pyramid
Demi-pyramid junction (Bump)	Simple central suture [16]	-	Bump between two demi-pyramids

In regular echinoids, the two demi-pyramids meet at a central suture and are not connected by any other skeletal structures [16]. In *S. mirabilis* possessed a distinct bump at the junction between the two demi-pyramids, which was connected to the demi-pyramids by the symphysis. Based on its position, this structure may contribute to reinforcement of the demi-pyramid junction.

Therefore, the Aristotle’s lantern of *S. mirabilis* exhibits morphological and microstructural specializations. These features provide new anatomical information on the lantern of this species and offer a morphological basis for future studies of feeding function and evolutionary adaptations in irregular echinoids.

## 5. Conclusions

In this study, the external morphology of *Scaphechinus mirabilis* and the micromorphology of Aristotle’s lantern were examined using light microscopy, scanning electron microscopy, and X-ray microscopy. Aristotle’s lantern consisted of the pyramids, teeth, epiphyses, rotula, ligaments, and muscles, whereas the compass and perignathic-lantern joint were absent. The pyramids were interconnected by connective tissue, and the teeth were arranged parallel to the body axis along the tooth grooves of the pyramids. X-ray microscopy further revealed the three-dimensional arrangement of these components within Aristotle’s lantern. These findings provide detailed morphological information on the structural organization of Aristotle’s lantern in *S. mirabilis* and contribute to understanding the micromorphological characteristics of the feeding apparatus in sand dollars.

## Figures and Tables

**Table 1:** Morphometric characteristics of *Scaphechinus mirabilis.*

Measurement	Mean ± SD	Range (Min–Max)
test length (mm)	40.77 ± 5.03	33.38–63.06
test height (mm)	5.20 ± 0.84	4.14–8.83
total weight (g)	6.45 ± 4.04	3.41–26.11

## Data Availability

The original contributions presented in this study are included in the article. Further inquiries can be directed to the corresponding authors.

## References

[1] Kroh A., Mooi R. World Echinoidea Database. https://www.marinespecies.org/echinoidea.

[2] Durham J.W., Moore R.C. (1966). Classification. Treatise on Invertebrate Paleontology. Part U. Echinodermata 3.

[3] WoRMS Editorial Board WoRMS Taxon Details: *Scaphechinus mirabilis* (AphiaID: 342067). https://www.marinespecies.org/aphia.php?p=taxdetails&id=342067.

[4] Kashenko S.D. (2008). Responses of the sand dollar *Scaphechinus mirabilis* to extreme environmental changes. Russ. J. Mar. Biol..

[5] Matsuda H., Hamano T., Nagasawa K. (2010). Spatial distribution patterns of the parasitic gastropod *Hypermastus tokunagai* (Eulimidae) in populations of its echinoid host *Scaphechinus mirabilis* (Scutellidae). Venus.

[6] Ho S.L., Wang J.K., Lin Y.J., Lin C.R., Lee C.W., Hsu C.H., Chang L.Y., Wu T.H., Tseng C.C., Wu H.J. (2022). Changing surface ocean circulation caused the local demise of echinoid *Scaphechinus mirabilis* in Taiwan during the Pleistocene–Holocene transition. Sci. Rep..

[7] National Marine Biodiversity Institute of Korea Marine Bio-Resource Information System (MBRIS). https://www.mbris.kr/pub/main/publicMainPage.do.

[8] Ageenko N.V., Kiselev K.V., Odintsova N.A. (2022). Quinoid pigments of sea urchins *Scaphechinus mirabilis* and *Strongylocentrotus intermedius*: Biological activity and potential applications. Mar. Drugs.

[9] Levin V.S., Korobkov V.A. (2003). Morskie Ezhi Rossii. Biologiya, Promysel, Ispol’zovanie.

[10] Rolet G., Ziegler A., De Ridder C. (2012). Presence of a seawater-filled caecum in *Echinocardium cordatum* (Echinoidea: Spatangoida). J. Mar. Biol. Assoc. UK.

[11] Kang D.-H. (1999). Ecological Characteristics and Growth of the Sand Dollar, *Astriclypeus manni* (VERRIL 1867). Master’s Thesis.

[12] Mooi R. (1990). Paedomorphosis, Aristotle’s lantern, and the origin of the sand dollars (Echinodermata: Clypeasteroida). Paleobiology.

[13] Perricone V., Grun T.B., Marmo F., Langella C., Candia Carnevali M.D. (2021). Constructional design of echinoid endoskeleton: Main structural components and their potential for biomimetic applications. Bioinspir. Biomim..

[14] Ziegler A., Schröder L., Ogurreck M., Faber C., Stach T. (2012). Evolution of a novel muscle design in sea urchins (Echinodermata: Echinoidea). PLoS ONE.

[15] Telford M., Ellers O. (1997). Tooth advancement muscles in the sand dollar *Echinarachnius parma*. Invertebr. Biol..

[16] Märkel K. (1979). Structure and growth of the cidaroid socket-joint lantern of Aristotle compared to the hinge-joint lanterns of non-cidaroid regular echinoids (Echinodermata, Echinoidea). Zoomorphology.

[17] Black R., Codd C., Hebbert D., Vink S., Burt J. (1984). The functional significance of the relative size of Aristotle’s lantern in the sea urchin *Echinometra mathaei* (de Blainville). J. Exp. Mar. Biol. Ecol..

[18] Killian C.E., Metzler R.A., Gong Y., Churchill T.H., Olson I.C., Trubetskoy V., Christensen M.B., Fournelle J.H., De Carlo F., Cohen S. (2011). Self-sharpening mechanism of the sea urchin tooth. Adv. Funct. Mater..

[19] Ziegler A., Gilligan A.M., Dillon J.G., Pernet B. (2020). Schizasterid heart urchins host microorganisms in a digestive symbiosis of mesozoic origin. Front. Microbiol..

[20] Uğurlu E. (2023). Characterization of shell, spine and Aristotle’s lanterns of the invasive *Diadema setosum* in Iskenderun Bay. Mau. J. Agr. Nat..

[21] Seilacher A. (1979). Constructional morphology of sand dollars. Paleobiology.

[22] Ellers O., Telford M. (1997). Muscles advance the teeth in sand dollars and other sea urchins. Proc. R. Soc. B-Biol. Sci..

[23] Sakurai I., Abe H., Ogata T. (2019). Reproductive cycle and growth of the sand dollar *Scaphechinus griseus* inhabiting Japanese surf clam *Pseudocardium sachalinense* fishing grounds in Tomakomai, Southwest Hokkaido, Japan. Fish. Sci..

[24] Ma Y., Aichmayer B., Paris O., Fratzl P., Meibom A., Metzler R.A., Politi Y., Addadi L., Gilbert P.U.P.A., Weiner S. (2009). The grinding tip of the sea urchin tooth exhibits exquisite control over calcite crystal orientation and Mg distribution. Proc. Natl. Acad. Sci. USA.

[25] Hewson I., Ritchie I.T., Evans J.S., Altera A., Behringer D., Bowman E., Brandt M., Budd K.A., Camacho R.A., Cornwell T.O. (2023). A scuticociliate causes mass mortality of *Diadema antillarum* in the Caribbean Sea. Sci. Adv..

[26] Kim H.J., Park J.J., Lee J.S. (2025). Transformation of sperm structure in *Octopus vulgaris*: From spermatogenesis to spermatophoric release. PLoS ONE.

[27] Cognigni F., Dinarelli S., Girasole M., Longo G., Fabi G., Rossi M. (2022). 3D X-Ray Microscopy (XRM) investigation of exogenous materials inside mussels’ organs. IOP Conf. Ser. Mater. Sci. Eng..

[28] Kier P.M. (1982). Rapid evolution in echinoids. Palaeontology.

[29] Telford M., Mooi R., Ellers O. (1985). A new model of podial deposit feeding in the sand dollar, *Mellita quinqujesperforata* (Leske): The sieve hypothesis challenged. Biol. Bull..

[30] Pomory C.M., Robbins B.D., Lares M.T. (1995). Sediment grain size preference by the sand dollar *Mellita tenuis* Clark, 1940 (Echinodermata: Echinoidea): A laboratory study. Bull. Mar. Sci..

[31] Timko P.L. (1976). Sand dollars as suspension feeders: A new description of feeding in *Dendraster excentricus*. Biol. Bull..

[32] Ellers O., Telford M. (1992). Causes and consequences of fluctuating coelomic pressure in sea urchins. Biol. Bull..

[33] Bonasoro F., Candia Carnevali M.D., Wilkie I.C. (1995). The peristomial membrane of regular sea-urchins: Functional morphology of the epidermis and coelomic lining in *Paracentrotus lividus* (Lamarck). Ital. J. Zool..

[34] Wilkie I.C., Candia Carnevali M.D., Andrietti F. (1994). Microarchitecture and mechanics of the sea-urchin peristomial membrane. Ital. J. Zool..

[35] Vellutini B.C., Migotto A.E. (2010). Embryonic, larval, and juvenile development of the sea biscuit *Clypeaster subdepressus* (Echinodermata: Clypeasteroida). PLoS ONE.

[36] Schultz H. (2006). Sea Urchins: A Guide to Worldwide Shallow Water Species.

[37] Candia Carnevali M.D., Wilkie I.C., Lucca E., Andrietti F., Melone G. (1993). The Aristotle’s lantern of the sea-urchin *Stylocidaris affinis* (echinoida, cidaridae): Functional morphology of the musculo-skeletal system. Zoomorphology.

[38] Shapkin N.P., Khalchenko I.G., Panasenko A.E., Drozdov A.L. (2017). Sea urchin skeleton: Structure, composition, and application as a template for biomimetic materials. Proceedings of the AIP Conference Proceedings.

[39] Dixon-Anderson I.S., Smith A.M. (2025). Detailed controls on biomineralization in an adult echinoderm: Skeletal carbonate mineralogy of the New Zealand sand dollar (*Fellaster zelandiae*). Biogeochemistry.

[40] Carnevali M.D.C., Bonasoro F., Melone G. (1991). Microstructure and mechanical design in the lantern ossicles of the regular sea-urchin *Paracentrotus lividusi* a scanning electron microscope study. Boll. Zool..

[41] Andrietti F., Candia Carnevali M.D., Wilkie I.C. (1993). A biomechanical comparison of the lantern of the cidarid sea-urchin *Stylocidaris affinis* with the typical camarodont lantern. J. Zool..

[42] Stock S.R. (2014). Sea urchins have teeth? A review of their microstructure, biomineralization, development and mechanical properties. Connect. Tissue Res..

[43] Challener R., Miller M., Furbish D., McClintock J. (2009). Evaluation of sand grain crushing in the sand dollar *Mellita tenuis* (Echinoidea: Echinodermata). Aquat. Biol..

[44] Chen C.-P., Lawrence J.M. (1986). The ultrastructure of the plumula of the tooth of *Lytechinus variegatus* (Echinodermata: Echinoidea). Acta Zool..

[45] Narvaez C.A., Padovani A.M., Stark A.Y., Russell M.P. (2020). Plasticity in the purple sea urchin (*Strongylocentrotus purpuratus*): Tube feet regeneration and adhesive performance. J. Exp. Mar. Biol. Ecol..

[46] Kroh A., Smith A.B. (2010). The phylogeny and classification of post-palaeozoic echinoids. J. Syst. Palaeontol..

